# The National Association of Dental Faculties

**Published:** 1893-09

**Authors:** 


### The National Association of Dental Faculties.

The National Association of Dental Faculties met in tenth annual session in Kindergarten College Hall, Chicago, August 10, 1893, the President, Dr. J. D. Patterson, presiding.

Delegates from twenty-two colleges were present at the first roll call.

Dr. Jas. Truman, Dental Department of the University of Pennsylvania, offered a resolution that but one delegate from each college be given the privilege of speaking, voting or acting on committees. Carried.

A recommendation from the Executive Committee to the effect "That all applications for membership must be endorsed by two or more members of the association" was adopted to take effect in 1894.

The application for membership of the Western Dental College, of Kansas City, was again laid over for a year.

Dr. L. D. Carpenter, Dental Department Southern Medical College, Atlanta, Ga., requested a ruling upon the by-law in regard to dissections. The president pronounced the language mandatory.

Dr. W. H. Morgan, Dental Department Vanderbilt University, reported that they had abandoned the preliminary course, substituting a practical course at the end of the session.

Dr. W. X. Sudduth, College of Dentistry, Department of Medicine, University of Minnesota, reported a preliminary quiz course for conditioned students; also that they had changed the degree from "D.D.S." to "D.M.D."

Dr. Goddard, Dental Department University of California, reported various changes in the curriculum, including the addition of Latin to the entrance examination, the elements of pharmacy for first-course students, a practical course in orthodontia for seniors, etc.

The applications for membership of the University of Buffalo, Dental Department, and the Western Reserve University, Dental Department, Cleveland, Ohio, under the rules lie over to next year.

A resolution offered last year by Dr. R. B. Winder admitting graduates in pharmacy to advanced standing was lost.

A similar resolution offered by Dr. Peirce, last year, was then taken up, amended, and adopted as follows:

"*Resolved*, That colleges of this association may admit to the junior class graduates of recognized schools of pharmacy, subject to the examinations of the freshman year."

The amendment to Article VII of the Constitution, offered last year, was on motion, laid on the table.

The following resolution, laid over from last year, was adopted:

"*Resolved*, That any college of this association failing to have a representative present for two consecutive years, without satisfactory explanation, shall be dropped from the roll of the association."

A motion of Dr. Sudduth that Latin and physics be added to the list for examination for entrance to the colleges belonging to the association lies over to next year.

On the favorable recommendation of the Executive Committee the Dental Department of the Detroit College of Medicine was elected to membership.

A similar recommendation of the Dental Department of the Homeopathic Hospital College, Cleveland, Ohio, was rejected and referred back to the committee, which reported adversely after further investigation, and the application was rejected.

The application of the Dental Department of the Howard University, Washington, D. C., lies over another year.

Adverse report upon the United States Dental College was unanimously adopted. A vote of censure upon Dr. Sudduth for language used reflecting upon a certain dental college, recommended by a special committee appointed to investigate the matter, was voted down on alleged lack of jurisdiction by the association.

A number of other resolutions were offered, which lay over one year.

The following resolution was adopted :

"*Resolved*, That a committee be appointed to formulate a series of subjects and questions for preliminary examinations and a minimum standard to be reached before admitting students to colleges."

Drs. Francis Peabody, W. X. Sudduth and H. W. Morgan were appointed on this special committee.

The election of officers resulted as follows:—President, Dr. H. A. Smith, Cincinnati; Vice-President, C. L. Goddard, San Francisco; Secretary, J. E. Cravens, Indianapolis; Treasurer, H. W. Morgan, Nashville.

Executive Committee.—Drs. A. O. Hunt, J. Taft, Frank Abbott.

Ad Interim Committee.—Drs. Jas. Truman, Thos. Fillebrown, W. H. Eames.

The officers elected were then installed and the association adjourned to meet at the call of the Executive Committee.

